# The Nitric Oxide Synthase Inhibitor N^G^-Nitro-L-Arginine Methyl Ester Diminishes the Immunomodulatory Effects of Parental Arginine in Rats with Subacute Peritonitis

**DOI:** 10.1371/journal.pone.0151973

**Published:** 2016-03-23

**Authors:** Hui-Chen Lo, Ching-Yi Hung, Fu-Huan Huang, Tzu-Cheng Su, Chien-Hsing Lee

**Affiliations:** 1 Department of Nutritional Science, Fu Jen Catholic University, New Taipei City, Taiwan; 2 Division of General Pediatric Surgery, Department of Surgery, China Medical University Children's Hospital, Taichung, Taiwan; 3 Department of Pathology, Changhua Christian Hospital, Changhua, Taiwan; 4 School of Chinese Medicine, College of Chinese Medicine, China Medical University, Taichung, Taiwan; University of Kentucky, UNITED STATES

## Abstract

The combined treatment of parenteral arginine and the nitric oxide synthase inhibitor N^G^-nitro-L-arginine methyl ester (L-NAME) have been shown to improve liver function and systemic inflammation in subacute peritonitic rats. Here, we investigated the effects of single and combined parenteral arginine and L-NAME treatments on leukocyte and splenocyte immunity. Male Wistar rats were subjected to cecal punctures and were intravenously given total parenteral nutrition solutions with or without arginine and/or L-NAME supplementations for 7 days. Non-surgical and sham-operated rats with no cecal puncture were given a chow diet and parenteral nutrition, respectively. Parenteral feeding elevated the white blood cell numbers and subacute peritonitis augmented the parenteral nutrition-induced alterations in the loss of body weight gain, splenomegaly, and splenocyte decreases. Parenteral arginine significantly increased the B-leukocyte level, decreased the natural killer T (NKT)-leukocyte and splenocyte levels, alleviated the loss in body weight gain and total and cytotoxic T-splenocyte levels, and attenuated the increases in plasma nitrate/nitrite and interferon-gamma production by T-splenocytes. L-NAME infusion significantly decreased NKT-leukocyte level, tumor-necrosis factor (TNF)-alpha production by T-splenocytes and macrophages, and interferon-gamma production by T-leukocytes, monocytes, and T-splenocytes, as well as increased interleukin-6 production by T-leukocytes and monocytes and nitrate/nitrite production by T-leukocytes. Combined treatment significantly decreased plasma nitrate/nitrite, the NKT-leukocyte level, and TNF-alpha production by T-splenocytes. Parenteral arginine may attenuate immune impairment and L-NAME infusion may augment leukocyte proinflammatory response, eliminate splenocyte proinflammatory and T-helper 1 responses, and diminish arginine-induced immunomodulation in combined treatment in subacute peritonitic rats.

## Introduction

Peritonitis, including the severe and subacute forms, has been regarded as an alternative arginine-deficient state [1]. Peritonitis-associated arginine deficiency, chronic inflammation, and dysfunctional immune system result in increased disease susceptibility and mortality [2]. Numerous studies have investigated the effects of arginine, nitric oxide donors, and nitric oxide synthase inhibitors in different sepsis-associated diseases [3–5]. However, subacute peritonitis has easily been overlooked because of its less severe clinical symptoms, such as, a cloudy effluent, abdominal pain, and fever [6].

Evidence showed that exogenous arginine may be a useful adjuvant in decreasing the mortality rate of septic patients by improving microcirculation, maintaining immune function, and alleviating oxidative stress [7]. Controversial results indicated that uncontrolled and excessive production of arginine-derived nitric oxide (NO), which is catalyzed by inducible nitric oxide synthase (NOS), may contribute to the oxidative and nitrasative stress, hemodynamic instability, cardiodepression, and vascular hyporeactivity observed in septic shock [4]. Considering the importance of NO in a variety of biological processes, partial inhibition of NOS activity might have beneficial effects in preserving the balance between the arginine-NO axis in sepsis [8]. Until now, the use of NO donors and NOS inhibitors in sepsis, including peritonitis, is still under investigation.

A number of patients with peritonitis need total parenteral nutrition (TPN) solutions to support their nutrition requirements; however, TPN support is an immunosuppressive and bacterial susceptible therapy [9]. In an acute peritonitic rat model with a 50% survival rate, the immunomodulatory effects of pretreatment with an arginine-supplemented TPN solution had been reported without improving survival [10]. In a subacute peritonitic rat model with a 100% survival rate, post-treatment with an arginine-supplemented TPN solution may enhance the systemic and splenocytic immunity [11]. However, the pharmacological dose of parenteral arginine, i.e., over 6% of the total calories, may not have benefits in anabolism and immunity [11,12]. In preventing excess NO production, the beneficial effects of N^G^-nitro-L-arginine methyl ester (L-NAME), a non-selective NOS inhibitor, have been shown in acute peritonitic mice [13], zinc deficient rats [14], and piglets with pneumonia and sepsis [15]. In contrast, intrarenal L-NAME infusion may not facilitate creatinine clearance in sheep with mild non-hypotensive and severe hypotensive sepsis [16]. In rats with subacute peritonitis and parenteral nutrition, intravenous L-NAME infusion may not alter systemic NO homeostasis and inflammatory responses; however, it may facilitate nitrogen excretion and arginine-associated amino acid production [17]. These contradictory results imply that disease severity may be an indicator for the usage of arginine and L-NAME treatments.

The effects of combined arginine and NOS inhibitor treatments on NO homeostasis and tissue injury have been investigated in rats with hind leg ischemia and reperfusion [18] and in rats with subacute peritonitis and parenteral nutrition [19]. The results showed that a single L-arginine treatment led to decreased inflammation and intestinal oxidative stress as well as improved liver function, whereas the combined treatment of L-arginine and L-NAME had similar but partially reversed effects as the single treatment of L-arginine [18,19]. In the present study, we further investigated the effects of single and combined parenteral arginine and L-NAME treatments on leukocyte and splenocyte immunity in rats with subacute peritonitis and parenteral nutrition. We hypothesized that parenteral arginine treatment is more beneficial than L-NAME in alleviating sub-acute peritonitis-induced immune impairment.

## Materials and Methods

### Animals and experimental design

This study was approved by the Laboratory Animal Care and Use Committee of Changhau Christian Hospital, Changhua, Taiwan (CCH-AE-92008). Male Wistar rats (LASCO Taiwan Co., Taipei, Taiwan), which were 5 to 6 weeks old and initially weighed 200 to 250 grams, were acclimated to animal facilities in a room that was maintained at 22°C on a 12:12-h light-dark cycle, and they had free access to water and chow diet for 1 week before surgery.

After an overnight fast, the animals were anesthetized with an intramuscular injection containing 80 mg/kg of ketamine and 8 mg/kg of xylazine (day 0). Once the animals lost consciousness, two surgeries were performed, including the placement of catheters in the superior vena cava by way of the external jugular vein for the infusion of parenteral nutrition solutions and the cecal puncture procedure involved cecum exposure, two punctures, and the extrusion of fecal materials for the induction of a subacute peritonitis as described in previous studies [12,20]. Normal control animals (R group, n = 8) that did not receive the cecal puncture procedure had free access to water and rat chow during the experimental period.

Forty-eight animals that underwent surgery were randomly assigned into treatment groups, which included conventional parenteral nutrition solution (CPP group, arginine 0.95 g/kg/day), parenteral nutrition supplemented with arginine (arginine 1.88 g/kg/day, ARG group), L-NAME (NAM group, L-NAME 25 mg/kg/day) or arginine plus L-NAME (COM group, arginine 1.88 g/kg/day and L-NAME 25 mg/kg/day). Twelve animals that underwent a sham cecal operation and were infused with conventional parenteral nutrition were included in the non-peritonitic and parenteral feeding control group (TPN group). The administered arginine (1.88 g/kg/day) and L-NAME (25 mg/kg/day) doses were based on results from our previous studies [12,20]. Several animals were excluded from the study because of catheter dislodgement, fluid extravasations, or thrombus incidences at the tip of the catheter. Therefore, the final sample sizes were 10, 10, 10, 11, and 12 rats in the TPN, CPP, ARG, NAM and COM groups, respectively.

### Composition of the TPN solutions

During the experimental period, the parenterally fed rats merely received nutrition from the parenteral nutrition solutions via intravenous infusion. The infusion amount was increased gradually from 25 kcal on day 0 to 62 kcal (250 kcal/kg/day) on days 1 to 6, as suggested by the study of Tao et al. [21] The parenteral nutrition solution contained amino acids (42 g/l, 10% Aminosyn with or without arginine, Abbott Laboratories, IL, USA), dextrose (160 g/l, 50% Paren-aid, Taita NO. V, Jungli, Taiwan) and a lipid emulsion (35 g/l, 20% Lipovenös^®^, Fresenius AG, D-6380 Bad Homburg v.d.H., Federal Republic of Germany). For the parenteral nutrition solutions without arginine supplementation, 10% Aminosyn was used to provide equal amounts of calories and protein in the 4 different parenteral nutrition solutions. For the arginine-supplemented solutions, the amount of arginine (1.88 g/kg/day) accounted for 3.24% of the total calories, which was approximately 2-fold more than the amount of arginine used in conventional parenteral nutrition solutions (0.95 g/kg/day), i.e., 1.61% of the total calories.

### Experimental procedures

To avoid interactions between the medicine and subacute peritonitis, the animals were not administered any antibiotics or anti-inflammatory drugs during the experimental period. The body weights and the infused amounts of parenteral nutrition solutions were recorded daily. Seven days after the parenteral nutrition infusion, the animals were killed under anesthesia with an intramuscular injection of 150 mg/kg ketamine and 15 mg/kg xylazine. The order of euthanasia was randomized among the groups. Blood was collected by cardiac puncture, after which the plasma and whole blood samples were isolated for further assays. The spleens were dissected, weighed, and processed for further analyses.

#### Circulating white blood cells (WBCs), plasma cytokines, and serum nitrate/nitrite

The number of WBCs was determined using a hematology analyzer (GEN, Coulter Inc., Miami, FL). Plasma concentrations of tumor necrosis factor (TNF)-α, interleukin (IL)-6, interferon (IFN)-γ and IL-4 were measured with commercially available enzyme-linked immunosorbent assays (ELISA; R&D Systems Inc., Minneapolis, MN and Mercodia AB, Uppsala, Sweden). Serum concentrations of nitrate/nitrite were measured using the Griess reaction [22]. All of the samples were analyzed in duplicate with inter-assay coefficients of variance below 10%.

#### Immunocyte subpopulations in the peripheral blood leukocytes and splenocytes

To determine the immunocyte subpopulations, whole blood and splenocytes (1 x 10^6^ cells) that were prepared from single-cell suspensions were incubated with antibodies against cell-surface antigens and analyzed by flow cytometry, as described in previous studies [17]. The cell-surface markers included CD3-fluorescein isothiocyanate (FITC, clone G4.18), CD3-phycoerythrin (PE, clone G4.18), CD4-PE (clone OX-35), and CD8b-FITC (clone 341) for T cells; CD45RA-PE (clone OX-33) and IgM-FITC (clone G53-238) for B cells; CD11b/c-PE (clone OX-42) for monocyte/macrophage; HIS48 for granulocytes-FITC (clone HIS48); and NKR-P1A-PE (clone 10/78) for natural-killer cells. FITC-conjugated mouse IgG1 (clone A112-2) and IgG2 (clone G155-178) and PE-conjugated mouse IgG3 (clone A112-3) were used as non-specific isotype control antibodies. Immunofluorescence detection of the cell subsets were performed using a Becton-Dickinson FASCScan flow cytometer with excitation capabilities at 488 nm for the measurements of the FITC- and PE-conjugated mouse anti-rat monoclonal antibodies (BD Biosciences, San Jose, CA, USA).

#### Immunocyte proliferation and cytokine and nitrate/nitrite production

To assess the immunocyte functions, the splenocyte proliferation ability and the leukocyte and splenocyte cytokine production abilities were determined. The *in vitro* proliferation of splenocytes in response to a T-cell mitogen, i.e., concanvalin A (Con A), and a B-cell and macrophage/monocyte mitogen, i.e., lipopolysaccharide (LPS), were measured. The splenocytes (2.5 x 10^5^ cells) were incubated in RPMI 1640 medium with or without Con A (5 μg/ml) and LPS (10 μg/ml) at 37°C in 5% CO_2_. After a 36-hour incubation, cell proliferation was determined by the MTS method (Promega, WI, USA). Using a spectrophotometer to obtain the absorbances at 490 nm, the stimulation index of cell proliferation was calculated from the splenocytes cultured with the mitogen-supplemented RPMI 1640 medium divided by those with RPMI 1640 medium alone.

The leukocytes and splenocytes (5 x 10^6^ cells) were incubated with RPMI 1640 medium with or without Con A (5 μg/ml) and LPS (10 μg/ml) at 37°C in 5% CO_2_. After 18 hours of incubation, the supernatants were removed and stored at -80°C for further analysis. The TNF-α, IFN-γ and IL-6 production levels from the leukocytes and splenocytes were measured with ELISAs. The nitrate/nitrite production levels from the leukocytes and splenocytes were measured using the Griess reaction [22]. The samples were analyzed in one assay in duplicate.

### Statistical analysis

The differences among the groups were compared with one-way analysis of variance (ANOVA) using the SAS general linear models program (SAS Institute Inc., Cary, NC). The values were reported as the means ± SEM. When ANOVA indicated an overall significant group effect at P < .05, the protective least-significant difference (LSD) technique was used as a *post hoc* analysis to compare the differences between groups. To determine the main effects of arginine, L-NAME and the interactions of arginine and L-NAME, two-way ANOVA was used to analyze each parameter among the CPP, ARG, NAM, and COM groups.

## Results

### Parenteral arginine alleviated subacute peritonitis-induced decreases in body weight gain

The daily amounts of infused parenteral nutrition solutions were from 55 to 62 g on days 1 to 6 and were not significantly different among the TPN, CPP, ARG, NAM and COM groups. Body weights were not significantly different among the parenterally fed groups on the surgical day and first 2 postoperative days; however, their body weights were significantly lower on days 3 to 7 compared with the R group. When the body weight gain was calculated from day 0 to day 7, the TPN group had significantly lower body weight gains than the R group and the CPP group had even lower body weight gains than the TPN group (Table 1). In the peritonitic rats, for example, the CPP, ARG, NAM, and COM groups, the parenteral arginine significantly alleviated the decrease in body weight gain. For the WBC numbers, the TPN group had significantly greater numbers than the R group. There were no significant differences in the WBC numbers among the TPN, CPP, ARG, NAM, and COM groups.

**Table 1:** Body weight gain, relative spleen weights, and numbers of white blood cells and splenocytes in normal rats and in peritonitic rats parenterally supplemented with arginine and/or L-NAME^1^.

Group	BW gain	WBC	Spleen weight	Splenocytes
	g/7 days	10^3^/ml	g/kg BW	x10^7^ cells
R	42.8 ± 3.5	6.80 ± 0.20	0.24 ± 0.01	98.1 ± 10.8
TPN	10.8 ± 0.3*	8.93 ± 0.25*	0.35 ± 0.01*	76.3 ± 6.7*
CPP	4.8 ± 1.1^†^^b^	9.55 ± 0.14	0.42 ± 0.01^†^	55.3 ± 7.2^†^
ARG	10.4 ± 0.3^a^	9.04 ± 0.35	0.40 ± 0.01	68.8 ± 4.9
NAM	8.0 ± 0.5^ab^	7.90 ± 0.26	0.42 ± 0.02	69.2 ± 7.3
COM	7.5 ± 0.7^ab^	8.24 ± 0.21	0.42 ± 0.02	65.9 ± 8.1
Main effects of two-way ANOVA in the 4 peritonitic groups^2^				
Arginine	NS	NS	NS	NS
L-NAME	NS	NS	NS	NS
Interaction	NS	NS	NS	NS

^1^Values are represented as mean ± SEM, n = 10–12 for each group.

Symbol * represents the TPN group was significantly different from the R group and

symbol † represents the CPP group was significantly different from the TPN group (one-way ANOVA with least significant difference, *P* < 0.05). Superscript letters indicated significant differences among the CPP, ARG, NAM and COM groups. BW gain is the weight gain during the seven days of experiment. WBC, white blood cells; BW, body weight.

^2^Values of two-way ANOVA are p-values for main effects, such as arginine, L-NAME and interactions of arginine and L-NAME in the CPP, ARG, NAM and COM groups. NS, not significant.

### Parenteral nutrition and subacute peritonitis resulted in splenomegaly and decreased splenocyte numbers

The relative weights of the spleens, when corrected for body weight, were significantly increased in the TPN group compared with the R group and were further increased in the CPP group compared with the TPN group (Table 1). Neither arginine nor L-NAME had a significant impact on spleen weight. Additionally, the splenocyte numbers in the TPN group were significantly decreased compared with the R group and were further decreased in the CPP group compared with the TPN group. There were no significant differences in the splenocyte numbers among the groups with subacute peritonitis.

### L-NAME infusion increased the plasma cytokine levels and parenteral arginine decreased the serum nitrate/nitrite levels during subacute peritonitis

The plasma concentrations of TNF-α, IL-6, IFN-γ, and IL-4 and the serum concentrations of nitrate/nitrite are shown in Table 2. There were no significant differences in the plasma TNF-α, IL-6, and IL-4 levels between the TPN and R groups and between the CPP and TPN groups. The plasma IFN-γ and serum nitrate/nitrite levels were 25% lower and 150% greater, respectively, in the TPN group compared with the R group; however, they were not significantly different between the CPP and TPN groups. In the subacute peritonitic rats, the ARG group had significantly decreased plasma TNF-α levels compared with the NAM group and decreased plasma IFN-γ and IL-4 levels compared with the NAM and COM groups. The ARG and COM groups had significantly decreased serum nitrate/nitrite concentrations compared with the CPP and NAM groups. The results of 2-way ANOVA indicated that the L-NAME infusion was the main factor that increased the plasma TNF-α, IFN-γ, and IL-4 levels and parenteral arginine was the main factor that decreased the serum nitrate/nitrite levels in the peritonitic rats.

**Table 2:** Plasma concentrations of cytokines and nitrate/nitrite in normal rats and in peritonitic rats parenterally supplemented with arginine and/or L-NAME^1^.

Group	TNF-α	IL-6	IFN-γ	IL-4	nitrate/nitrite
	mg/l	mg/l	mg/l	mg/l	mmol/l
R	97.5 ± 6.8	367.9 ± 28.9	62.5 ± 4.3	15.0 ± 1.9	136.3 ± 10.2
TPN	95.4 ± 4.4	355.5 ± 11.5	46.4 ± 2.43	13.1 ± 1.1	337.4 ± 27.1*
CPP	88.6± 4.8^ab^	387.0 ± 19.0	52.6 ± 4.9^ab^	13.2 ± 1.7^ab^	375.6 ± 22.4^a^
ARG	84.1 ± 2.9^b^	378.2 ± 9.5	43.2 ± 2.4^b^	11.4 ± 0.9^b^	221.0 ± 14.8^b^
NAM	113.1 ± 15.1^a^	374.3 ± 8.3	69.4 ± 12.8^a^	16.4 ± 1.7^a^	354.9 ± 18.2^a^
COM	111.3 ± 10.5^ab^	349.8 ± 9.5	68.2 ± 9.6^a^	16.5 ± 1.3^a^	268.5 ± 15.9^b^
Main effects of two-way ANOVA in four peritonitic groups^2^					
Arginine	NS	NS	NS	NS	<0.001
L-NAME	0.014	NS	0.024	0.006	NS
Interaction	NS	NS	NS	NS	NS

^1^Values are represented as mean ± SEM, n = 10–12 for each group.

Symbol * represents the TPN group was significantly different from the R group (one-way ANOVA with least significant difference, *P* < 0.05).

Superscript letters indicated significant differences among the CPP, ARG, NAM and COM groups. TNF, tumor-necrosis factor; IFN, interferon; IL, interleukin.

^2^Values of two-way ANOVA are p-values for main effects, such as arginine, L-NAME and interactions of arginine and L-NAME in the CPP, ARG, NAM and COM groups. NS, not significant.

### Parenteral arginine increased mature B-leukocytes levels and L-NAME infusion decreased the NKT-leukocyte levels

There were no significant differences in the total T-cell, T-suppressor cell, granulocyte, and monocyte percentages among the groups in terms of PBLs (Table 3). The T-helper and NK non-T-leukocyte percentages were significantly decreased in the TPN group compared with the R group (*P* <0.05). There were no significant differences in the PBL subpopulation distributions between the TPN and CPP groups. However, the percentages of mature B-leukocytes were significantly increased in the ARG group compared with the CPP and COM groups and that of NKT-leukocytes were significantly decreased in the ARG, NAM, and COM groups compared to the CPP group. The results of the 2-way ANVOA indicated that arginine was a significant factor in increasing the mature B-leukocyte levels, whereas there was a significant interaction between arginine and L-NAME, that is, L-NAME diminished and even reversed the arginine effect in the combination treatment. Additionally, L-NAME was a significant factor in decreasing the NKT-leukocyte percentages.

**Table 3:** Percentages of immunocyte subsets in the peripheral blood in normal rats and in peritonitic rats parenterally supplemented with arginine and/or L-NAME^1^.

Group	CD3^+^	CD3^+^CD4^+^	CD3^+^CD8b^+^	CD45RA^+^IgM^+^	CD11b/c^+^HIS48^+^	CD11b/c^+^HIS48^-^	NKR-P1A^+^CD3^-^	NKR-P1A^+^CD3^+^
	%	%	%	%	%	%	%	%
R	50.0 ± 3.1	33.6 ± 1.6	25.6 ± 1.1	18.25 ± 2.75	37.28 ± 2.43	3.08 ± 1.11	5.97 ± 0.28	0.87 ± 0.16
TPN	45.1 ± 2.5	28.5 ± 1.7*	21.0 ± 3.9	20.12 ± 4.08	35.92 ± 2.17	4.34 ± 0.83	3.22 ± 0.77*	0.89 ± 0.07
CPP	45.0 ± 3.2	26.4 ± 1.9	17.9 ± 2.4	15.53 ± 2.50^bc^	32.46 ± 2.10	3.17 ± 0.69	3.21 ± 0.32	0.98 ± 0.10^a^
ARG	42.4 ± 1.0	25.8 ± 1.1	20.7 ± 1.1	23.24 ± 3.40^a^	34.54 ± 2.64	4.84 ± 0.97	3.66 ± 0.48	0.62 ± 0.13^b^
NAM	46.0 ± 1.5	24.9 ± 1.9	15.4 ± 2.3	21.70 ± 1.17^ab^	29.68 ± 1.61	6.09 ± 1.18	3.59 ± 0.51	0.47 ± 0.09^b^
COM	43.0 ± 1.4	25.7 ± 1.4	18.7 ± 1.3	13.63 ± 1.82^c^	28.80 ± 1.54	4.50 ± 0.94	3.66 ± 0.41	0.48 ± 0.04^b^
Main effects of two-way ANOVA in four peritonitic groups^2^								
Arginine	NS	NS	NS	0.034	NS	NS	NS	NS
L-NAME	NS	NS	NS	NS	NS	NS	NS	0.002
Interaction	NS	NS	NS	0.002	NS	NS	NS	NS

^1^Values are represented as mean ± SEM, n = 10–12 for each group.

Symbol * represents the TPN group was significantly different from the R group (one-way ANOVA with least significant difference, *P* < 0.05).

Superscript letters indicated significant differences among the CPP, ARG, NAM and COM groups. CD3+, total T cells; CD3+CD4+, T-helper cells; CD3+CD8b+, T-suppressor cells; CD45RA+IgM+, mature B cells; CD11b/c+HIS48+, granulocytes; CD11b/c+HIS48-, monocytes; NKR-P1A+CD3-, natural killer cells; NKR-P1A+CD3+, natural killer T cells.

^2^Values of two-way ANOVA are p-values for main effects, such as arginine, L-NAME and interactions of arginine and L-NAME in the CPP, ARG, NAM and COM groups. NS, not significant.

### Parenteral arginine increased the total T-cell and T-suppressor splenocyte percentages and decreased the NKT-splenocyte percentages

There were no significant differences in the T-helper splenocyte, mature B-splenocyte, granulocyte, and macrophage percentages among the groups in the spleen (Table 4). Compared with the R group, the TPN group had approximately 30%, 30%, 50%, and 40% decreases in the total T-, T-suppressor, NK non-T-, and NKT-splenocyte percentages, respectively. The CPP group had a further decrease in the total T-splenocyte percentages compared to the TPN group (*P* < 0.05). In subacute peritonitic rats, the ARG group had significantly elevated percentages in total T- and T-suppressor splenocytes and had further decreased percentages in NKT-splenocytes. The results of the 2-way ANOVA indicated that arginine was the main factor in increasing the total T- and T-suppressor splenocyte percentages and in decreasing the NKT-splenocyte percentages in the subacute peritonitic rats (*P* < 0.05).

**Table 4:** Percentages of immunocyte subsets in the splenocytes in normal rats and in peritonitic rats parenterally supplemented with arginine and/or L-NAME^1^.

Group	CD3^+^	CD3^+^CD4^+^		CD3^+^CD8b^+^		CD45RA^+^IgM^+^		CD11b/c^+^HIS48^+^		CD11b/c^+^HIS48^-^		NKR-P1A^+^CD3^-^		NKR-P1A^+^CD3^+^	
	%	%		%		%		%		%		%		%	
R	47.4 ± 2.3	28.1 ± 1.7		16.93 ± 1.02		6.93 ± 0.34		37.61 ± 2.46		17.35 ± 2.23		5.50 ± 0.25		1.21 ± 0.09	
TPN	32.76 ± 2.46*	23.1 ± 2.9		11.51 ± 1.06*		8.96 ± 0.78		33.70 ± 2.19		11.67 ± 0.59		2.68 ± 0.36*		0.71 ± 0.12*	
CPP	27.4 ± 1.3^†^^b^	18.5 ± 2.5		9.80 ± 0.50^b^		10.32 ± 0.94		32.83 ± 0.98		10.76 ± 1.17		3.02 ± 0.46		0.78 ± 0.09^a^	
ARG	33.5 ± 1.5^a^	17.6 ± 2.9		13.47 ± 0.79^a^		14.47 ± 4.01		28.63 ± 1.50		15.92 ± 4.47		3.20 ± 1.15		0.49 ± 0.11^b^	
NAM	25.2 ± 1.3^b^	21.5 ± 3.4		9.62 ± 0.49^b^		9.70 ± 0.85		31.90 ± 0.73		15.40 ± 0.99		2.16 ± 0.36		0.90 ± 0.08^a^	
COM	27.5 ± 1.2^b^	20.5 ± 2.6		8.96 ± 0.30^b^		10.96 ± 1.77		30.96 ± 2.43		14.44 ± 1.35		3.27 ± 0.42		0.78 ± 0.08^a^	
Main effects of two-way ANOVA in four peritonitic groups^2^															
Arginine	0.010		NS		0.009		NS		NS		NS		NS		0.006
L-NAME	NS		NS		NS		NS		NS		NS		NS		NS
Interaction	NS		NS		NS		NS		NS		NS		NS		NS

^1^Values are represented as mean ± SEM, n = 10–12 for each group.

Symbol * represents the TPN group was significantly different from the R group and

symbol † represents the CPP group was significantly different from the TPN group (one-way ANOVA with least significant difference, *P* < 0.05). Superscript letters indicated significant differences among the CPP, ARG, NAM and COM groups. CD3+, total T cells; CD3+CD4+, T-helper cells; CD3+CD8b+, T-suppressor cells; CD45RA+IgM+, mature B cells; CD11b/c+HIS48+, granulocytes; CD11b/c+HIS48-, macrophages; NKR-P1A+CD3-, natural killer cells; NKR-P1A+CD3+, natural killer T cells.

^2^Values of two-way ANOVA are p-values for main effects, such as arginine, L-NAME and interactions of arginine and L-NAME in the CPP, ARG, NAM and COM groups. NS, not significant.

### The L-NAME infusion decreased the T-splenocyte and splenic macrophage proliferation ability

**Fig 1:**
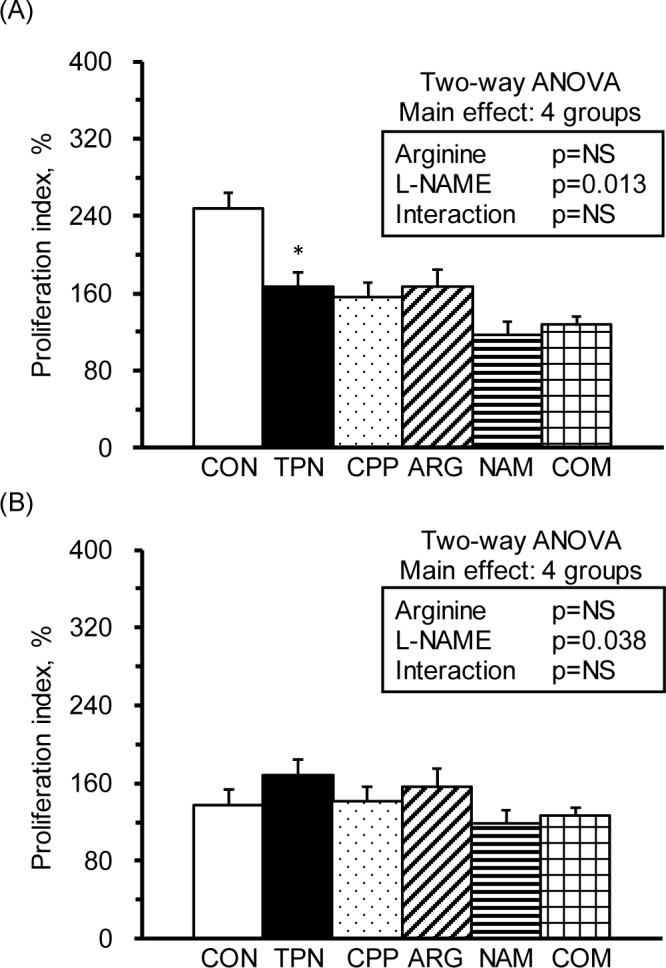
Cell proliferation of splenocytes. The stimulation indices of cell proliferation in splenocytes cultured with medium, Con A (A) and LPS (B). The stimulation index was calculated from OD values (at 490 nm) of splenocytes cultured with Con A or LPS divided by those with RPMI 1640 medium and multiplied by 100. The values are represented as the means ± SEM, n = 10–12 for each group. The * symbol represents that the TPN group was significantly different from the R group (one-way ANOVA with least significant difference, *P* < 0.05). The values from two-way ANOVA were p-values for the main effects, such as arginine, L-NAME and interactions of arginine and L-NAME, in the CPP, ARG, NAM and COM groups. NS, not significant.

To evaluate the T cell and macrophages, splenocytes were stimulated with the mitogens, Con A and LPS, respectively. The results showed that the TPN group had significantly a decreased proliferation ability regarding the T-splenocytes compared with the R group (Fig 1A). There were no significant differences in the proliferation abilities of splenic macrophage among groups (Fig 1B). However, the results of 2-way ANOVA revealed that L-NAME, not arginine, was the main factor in decreasing the proliferation abilities of T-splenocytes (Fig 1A insert) and splenic macrophages (Fig 1B insert).

### Parenteral arginine alleviated L-NAME-induced changes in T-leukocyte and monocyte cytokine and nitrate/nitrite production

**Fig 2:**
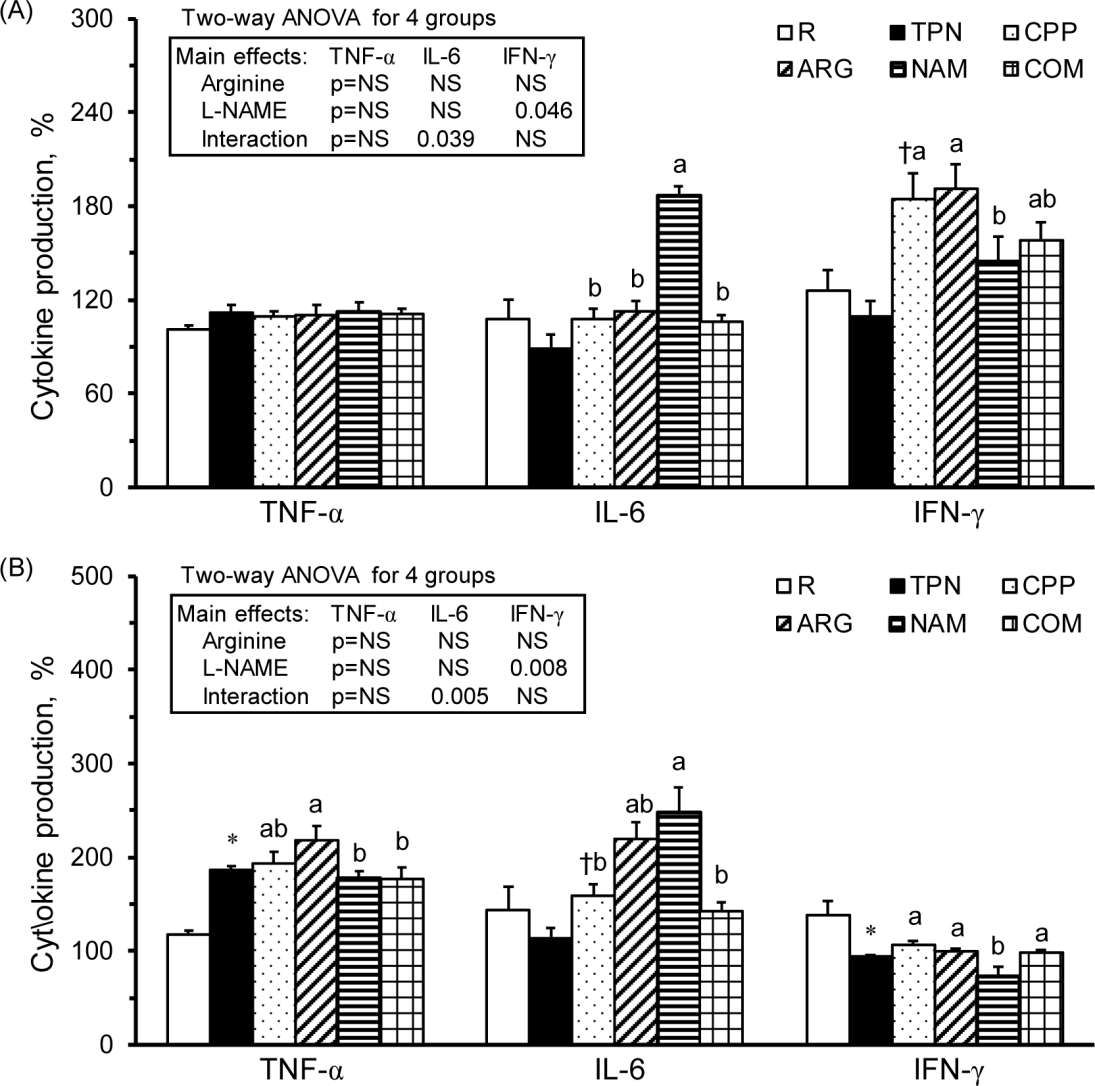
Cytokine productions of leukocytes. TNF-α, IL-6, and IFN-γ production from leukocytes cultured in medium with (A) Con A (5 mg/ml) and (B) LPS (10 mg/ml). The values were calculated from the cytokine production levels from leukocytes cultured with Con A or LPS divided by those with RPMI 1640 medium and multiplied by 100. The values are represented as the means ± SEM, n = 10–12 for each group. The * symbol represents that the TPN group was significantly different from the R group, and the † symbol represents that the CPP group was significantly different from the TPN group (one-way ANOVA with least significant difference, *P* < 0.05). The superscript letters indicate significant differences among the CPP, ARG, NAM and COM groups. The values from two-way ANOVA were p-values for the main effects, such as arginine, L-NAME and interactions of arginine and L-NAME, in the CPP, ARG, NAM and COM groups. NS, not significant.

To further evaluate the function of T-leukocytes and monocytes, cytokine production from mitogen-stimulated PBLs was determined. With Con A stimulation, the TNF-α, IL-6, and IFN-γ production levels were not significantly different between the TPN and R groups; however, the IFN-γ levels were significantly increased in the CPP group compared with the TPN group (Fig 2A). In the subacute peritonitic rats, the NAM group had significantly increased IL-6 production levels and decreased IFN-γ production levels from their T-leukocytes compared with the CPP group. The results of 2-way ANOVA indicated that L-NAME was the main factor in decreasing IFN-γ production in T-leukocytes. There was a significant interaction between arginine and L-NAME in IL-6 production (Fig 2A insert), that is, arginine significantly alleviated the effects of L-NAME on decreasing IL-6 production in the combination treatment.

Following LPS stimulation, the TPN group had significantly increased TNF-α production levels and decreased IFN-γ production levels from monocytes compared with the R group (Fig 2B). IL-6 production was significantly increased in the CPP group compared with the TPN group. Additionally, the NAM group had significantly increased IL-6 production and decreased IFN-γ production from monocytes compared with the CPP group. In the subacute peritonitic rats, L-NAME was the main factor in decreasing IFN-γ production in monocytes (Fig 2B insert). The significant interaction between arginine and L-NAME regarding IL-6 production indicated that both arginine and L-NAME were the main factors in regulating this cytokine; however, the effect of L-NAME in increasing IL-6 production in monocytes was abolished by arginine.

**Fig 3:**
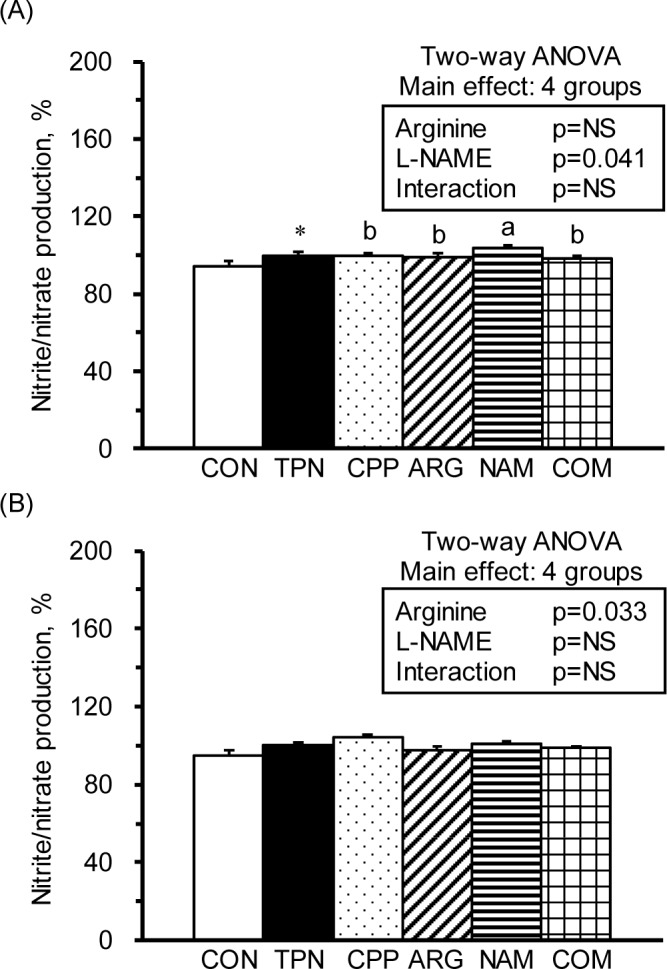
Nitrate/nitrite production of leukocytes. Nitrate/nitrite production from leukocytes cultured in medium with (A) Con A (5 mg/ml) and (B) LPS (10 mg/ml). The values were calculated from the nitrate/nitrite production levels of leukocytes cultured with Con A or LPS divided by those with RPMI 1640 medium and multiplied by 100. The values are represented as the means ± SEM, n = 10–12 for each group. The * symbol represents that the TPN group was significantly different from the R group (one-way ANOVA with least significant difference, *P* < 0.05). The superscript letters indicate significant differences among the CPP, ARG, NAM and COM groups. The values from two-way ANOVA were p-values for the main effects, such as arginine, L-NAME and interactions of arginine and L-NAME, in the CPP, ARG, NAM and COM groups. NS, not significant.

The nitrate/nitrite production levels from Con A-stimulated leukocytes, that is, T-leukocytes, were significantly increased in the TPN group compared with the R group (Fig 3A). In the subacute peritonitic rats, the NAM group had significantly increased nitrate/nitrite production compared with the CPP group. The production of nitrate/nitrite from LPS-stimulated leukocytes, that is, monocytes, was not significantly different among the groups (Fig 3B). However, the results of 2-way ANOVA indicated that L-NAME was the main factor in increasing the nitrate/nitrite production in T-leukocytes (Fig 3A insert) and arginine was the main factor in decreasing the nitrate/nitrite production in monocytes (Fig 3B insert) in the subacute peritonitic rats.

### The L-NAME infusion decreased cytokine and nitrate/nitrite production levels from T-splenocytes and parenteral arginine elevated the pro-inflammatory cytokine and nitrate/nitrite production levels from splenic macrophages

**Fig 4:**
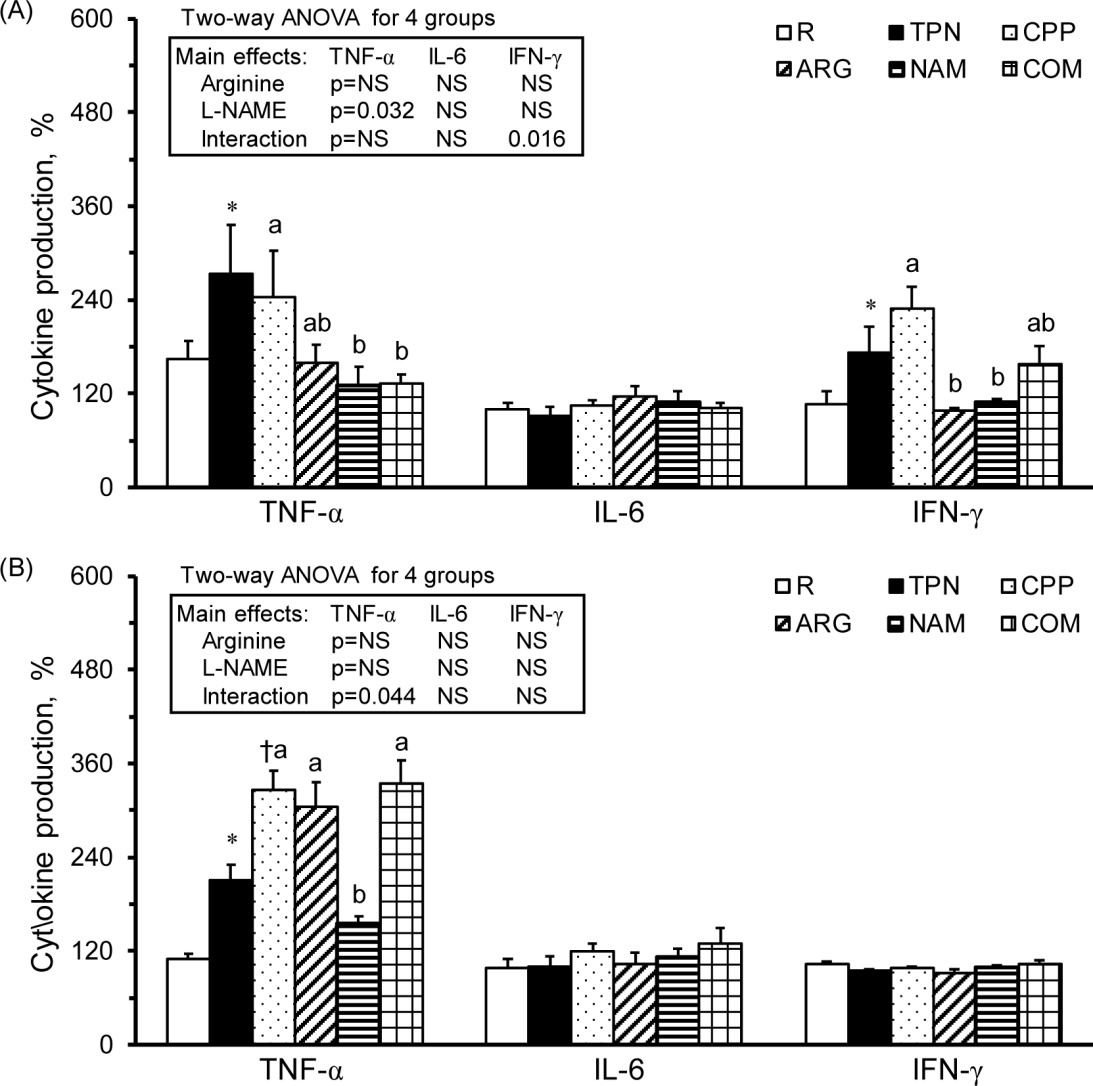
Cytokine productions of splenocytes. TNF-α, IL-6, and IFN-γ production from splenocytes (5 x 10^6^ cells) cultured in medium with (A) Con A (5 mg/ml) and (B) LPS (10 mg/ml). The values were calculated from the cytokine production levels from splenocytes cultured with Con A or LPS divided by those with RPMI 1640 medium and multiplied by 100. The values are represented as the means ± SEM, n = 10–12 for each group. The * symbol represents that the TPN group was significantly different from the R group, and the † symbol represents that the CPP group was significantly different from the TPN group (one-way ANOVA with least significant difference, *P* < 0.05). The superscript letters indicate significant differences among the CPP, ARG, NAM and COM groups. The values from two-way ANOVA were p-values for the main effects, such as arginine, L-NAME and interactions of arginine and L-NAME, in the CPP, ARG, NAM and COM groups. NS, not significant.

Following Con A stimulation, the TPN group had significantly increased TNF-α and IFN-γ production levels in their T-splenocytes (Fig 4A). The NAM and COM groups had significantly decreased TNF-α production and the ARG and NAM groups had significantly decreased IFN-γ production compared with the CPP groups regarding their T-splenocytes. There was no significant difference in the IL-6 production in T-splenocytes among the groups. The results of 2-way ANOVA indicated that L-NAME was the main factor in decreasing TNF-α production in splenic macrophages (Fig 4A insert). Additionally, the interaction between arginine and L-NAME revealed that both the arginine and L-NAME treatments were the main factor in decreasing the IFN-γ production from the splenic macrophages; however, the combined treatment may eliminate the effects of arginine and L-NAME on IFN-γ production.

Following LPS stimulation, the TPN group had significantly increased TNF-α production levels in macrophages compared with the R group, the CPP group had further increased production compared with the TPN group, and the NAM group had significantly decreased TNF-α production compared with the CPP group (Fig 4B). There were no significant differences in the IL-6 and IFN-γ production levels s in splenic macrophages among the groups. The results of 2-way ANOVA showed that there was a significant interaction between arginine and L-NAME regarding TNF-α production in splenic macrophages, and the effects of L-NAME in decreasing TNF-α production was alleviated in the combined treatment (Fig 4B insert). Neither arginine nor L-NAME was the main factor that altered the IL-6 and IFN-γ production from the splenic macrophages.

**Fig 5:**
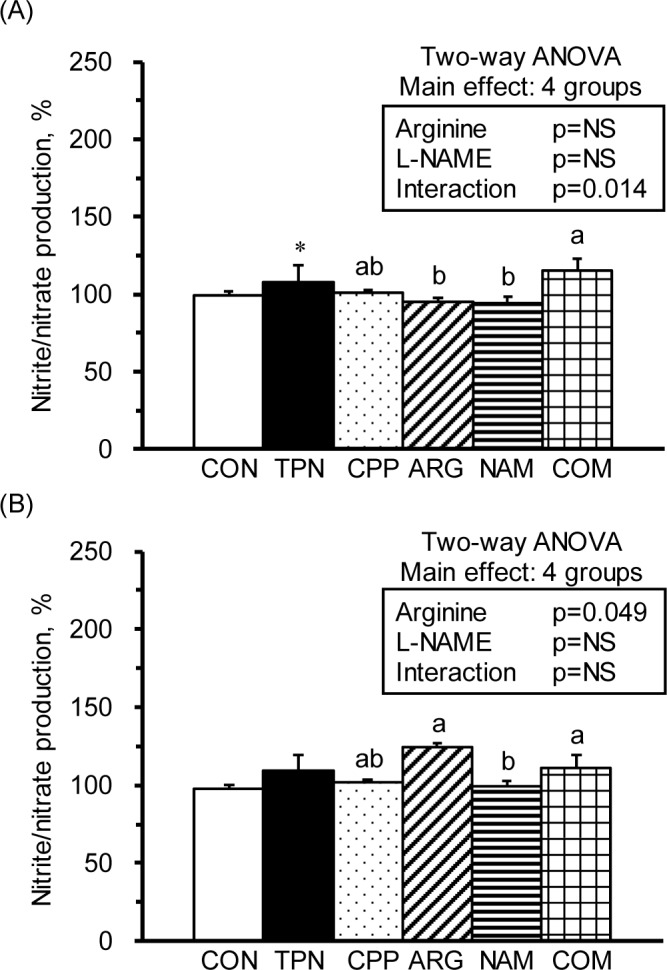
Nitrate/nitrite production of splenocytes. Nitrate/nitrite production from splenocytes (5 x 10^6^ cells) cultured in medium with (A) Con A (5 mg/ml) and (B) LPS (10 mg/ml). The values were calculated from the nitrate/nitrite production from splenocytes cultured with Con A or LPS divided by those with RPMI 1640 medium and multiplied by 100. The values are represented as the means ± SEM, n = 10–12 for each group. The * symbol represents that the TPN group was significantly different from the R group (one-way ANOVA with least significant difference, *P* < 0.05). The superscript letters indicate significant differences among the CPP, ARG, NAM and COM groups. The values from two-way ANOVA were p-values for the main effects, such as arginine, L-NAME and interactions of arginine and L-NAME, in the CPP, ARG, NAM and COM groups. NS, not significant.

In the T-splenocytes, the production of nitrate/nitrite was significantly increased in the TPN group compared with the R group and the COM group compared with the ARG and NAM groups (Fig 5A). The significant interaction between arginine and L-NAME indicated that the combined treatment might stimulate nitrate/nitrite production in macrophages in the spleen (Fig 5A insert). In the splenic macrophages, the nitrate/nitrite production in the ARG and COM groups was significantly increased compared with the NAM group (Fig 5B). The results of the 2-way ANOVA indicated that arginine was the main factor in increasing the nitrate/nitrite production from splenic macrophages (Fig 5B insert).

## Discussion

Controversial results regarding arginine supplementation have been shown in animals with severe sepsis [4,10]. Subacute peritonitis, a less severe peritonitis that is accompanied by immune dysfunction and relative arginine deficiency [12,19,20], has been regarded as an appropriate clinical condition to use arginine supplementation compared with severe peritonitis. Previous studies showed that parenteral arginine, at approximately 2 to 4% of the total calories, may have beneficial effects in facilitating ureagenesis as well as enhancing leukocytic and splenocytic responses, and it does not result in elevated nitrate/nitrite levels in parenteral-fed rats with subacute peritonitis [11,12]. Additionally, chronic infusion of L-NAME partially inhibits NOS activity, which may alleviate arginine deficiency and modulate the immunocytic response in a dose-dependent manner without altering systemic NO homeostasis and inflammatory responses [17,20]. When administered with a combined treatment of parenteral arginine and L-NAME, subacute peritonitic rats had improved liver function and alleviated inflammatory responses, and these beneficial effects may have been mainly due to parenteral arginine [19]. In the present study, we further demonstrated that parenteral arginine with or without L-NAME infusion may attenuate parenteral nutrition- and peritonitis-associated immune impairment and that the infusion of L-NAME may augment systemic pro-inflammatory responses and eliminate splenocytic pro-inflammatory and T-helper 1 responses.

### Parenteral nutrition may eliminate immunocyte numbers and elevate immunocyte responses

The immunosuppressive effects of parenteral nutrition have been noticed for years; however, its roles in improving malnutrition cannot be overlooked. Researchers are still working on modifying the components of parenteral nutrition to avoid adverse effects [23]. In the present study, we confirmed the immunomodulatory effects of a total parenteral nutrition solution. For example, the parenterally fed rats that were not subjected to subacute peritonitis had significantly increased circulating WBCs, spleen weights (Table 1), and serum nitrate/nitrite levels (Table 2), as well as significantly decreased splenocyte numbers and helper T-leukocyte, NK non-T-leukocyte (Table 3), cytotoxic T-splenocyte, NKT-splenocyte, and NK non-T-splenocyte percentages compared with healthy control rats (Table 4). Regarding immunocytic function, the total parenteral nutrition significantly decreased T-splenocytic proliferation (Fig 1A), increased nitrate/nitrite production from T-leukocytes (Fig 3A) and T-splenocyte (Fig 5A), and increased TNF-a production from monocytes (Fig 2B) and macrophages (Fig 4B). These results suggest that parenteral nutrition may elevate the systemic and splenocytic immune responses, whereas it may eliminate immunocyte populations, especially T and NK cells. Whether the elevated immune response is only an immune adaptation to compensate for the decreased numbers of immunocytes or a true immune enhancement requires further investigation.

### Subacute peritonitis activates the splenocyte response without increasing systemic inflammatory mediators

It has been indicated that patients with peritonitis, especially those with shock or non-survivors, have activated acute systemic innate immune responses, as evidenced by simultaneously increased plasma pro- and anti-inflammatory cytokines [24,25]. In parenteral-fed rats, we found that subacute peritonitis significantly decreased the splenocyte numbers (Table 1) and the T-splenocyte population (Table 4) and increased the production of pro-inflammatory cytokines in macrophages (Fig 4B); however, there were no significant changes in the plasma pro- and anti-inflammatory cytokines and serum nitrate/nitrite levels (Table 2). These results suggest that the subacute peritonitic rats with parenteral feeding may result in a local instead of systemic immune or cytokine storm as found in severe peritonitis.

### Parenteral arginine may attenuate inflammation and improve cellular immunity in subacute peritonitis

As shown in previous and present studies, subacute peritonitic rats with parenteral feeding had significantly decreased body weight gains, serum albumin levels, and plasma citrulline levels; however, they had significantly increased serum GOT, circulating WBC, insulin, inflammatory mediator (i.e., nitrate/nitrite, IL-6, prostaglandin E_2_), and glutamine levels [12,19,20]. These results indicate that these animals were under a catabolic and inflammatory state. Additionally, parenteral feeding and subacute peritonitis resulted in splenomegaly and decreased splenocyte numbers and proliferation capacity. When administered with parenteral arginine, the subacute peritonitic rats had reversed body weight gain (Table 1) and alleviated serum nitrate/nitrite levels, and they had increased B-leukocyte and cytotoxic T-splenocyte populations and decreased NKT-leukocyte and NKT-splenocyte populations. The decreased serum nitrate/nitrite and increased plasma ornithine levels revealed that parenteral arginine might activate arginase instead of NOS during subacute peritonitis. Unlike enteral arginine supplementation, which is utilized in promoting insulin resistance [26], parenteral arginine may be used as a fuel for whole body anabolism and cellular immune activation without causing hyperinsulinemia and inflammation. The results of two-way ANOVA suggested that parenteral arginine was the main factor in decreasing the serum nitrate/nitrite production levels, the monocyte nitrate/nitrite production levels (Fig 3B), and the NKT-splenocyte population. Additionally, parenteral arginine increased the B-leukocyte and T-splenocyte populations (Tables 3 and 4) and nitrate/nitrite production from the splenic macrophages (Fig 5B). These findings imply that parenteral arginine supplementation at 3.24% of the total calories may provide beneficial effects in improving weight gain, inflammation, and cellular immunity during subacute peritonitis.

### Daily treatment of L-NAME at a dose of 25 mg/kg may elevate systemic inflammatory response and attenuate splenocytic response in subacute peritonitis

The beneficial effect of L-NAME has been demonstrated in acute peritonitic rats infected with skin flora [14,27], newborn piglets with Escherichia coli and sepsis [15], and critically ill patients with severe refractory sepsis [28]. The L-NAME doses that were used in these studies were less than 24 mg/kg in a day. In bacterial challenged rats, intraperitoneal pretreatment of L-NAME at a dose of 100 mg/kg significantly increased neutrophil adhesion in the lung and decreased their adhesion in the peritoneum [29]. In subacute peritonitic rats with parenteral feeding, daily L-NAME infusion at doses of 5, 25, and 50 mg/kg may not alter systemic NO homeostasis or inflammation; however, they may decrease the nitrogen balance, facilitate the production of arginine-derived amino acids [20], and modulate adaptive and innate immunity in an dose- and tissue-dependent manner [17].

In the present study, the L-NAME infusion (25 mg/kg) significantly increased the IL-6 production from T-leukocytes and monocytes (Fig 2) and decreased TNF-α production in T-splenocytes and splenic macrophages (Fig 4). The results of two-way ANOVA showed that L-NAME was the main factor in increasing the plasma pro- and anti-inflammatory cytokines (Table 2) and the nitrate/nitrite production in T-leukocytes (Fig 3A); however, it decreased T-splenocyte and splenic macrophage proliferation (Fig 1) and IFN-γ production in T-leukocytes and monocytes (Fig 2). The unexpected increase in NOx production by T-leukocytes in the NAM group may be due to the non-inhibited NOS activity, since we did not add L-NAME in the RPMI-1640 medium. These studies reveal that L-NAME treatment in inflammatory diseases may result in both beneficial and adverse effects on hemodynamics and immunity, depending on the dose that is used.

### Parenteral arginine, instead of L-NAME has beneficial effects on alleviating inflammatory response and impaired immune responses in combined treatment in subacute peritonitis

It is proposed that combined arginine and L-NAME treatment may avoid overproduction of NO from NOS and stimulate the fuel utilization of arginine from arginase in inflammatory diseases. However, the application of combined arginine and L-NAME treatment is still debatable. For example, chronic administration of L-NAME provoked ascites-pulmonary hypertension syndrome and cardiac and pulmonary edema, and these changes were significantly improved by arginine treatment in chickens [30]. In an *ex vivo* study, arginine may induce jejunal fluid secretion via increased NO production. Additionally, a low dose of L-NAME reversed arginine-induced fluid secretion; however, a high L-NAME dose augmented these levels [31]. In rats with acute hind leg ischemia and reperfusion injury, arginine treatment had protective effects on alleviating tissue damages and lipid peroxidation [18]. In contrast, arginine pretreatment prolonged bleeding time and increased blood loss, and L-NAME with or without arginine reduced bleeding time and blood loss in rats with tail amputation [32]. No study has ever investigated the immunomodulatory effects of a combined parenteral arginine and L-NAME treatment. In the present study, we showed that parenteral arginine alleviated L-NAME-induced alterations in IL-6, IFN-γ (Fig 2), and nitrate/nitrite (Fig 3) production from T-leukocytes and monocytes, IFN-γ production from T-splenocytes, and TNF-α production from splenic macrophages (Fig 4). Based on the results of these studies, we speculate that parenteral arginine may be the major contributor in alleviating the inflammatory response, facilitating arginase activity, and reversing the impaired immune responses in rats with subacute peritonitis.

### Limitations of the present study

Several limitations are inherent in this study. First, for the local immune response, we only collected the spleen, not the thymus, gut-associated lymphoid tissues or bone marrow. The reasons that we chose the spleen instead of other tissues or organs were that the spleen is the body’s largest immune organ; therefore, it can provide enough immune cells for assays and has both innate and adaptive immune cells. Therefore, this study only investigated part of the immunity changes in the body. Second, we only investigated the immune response on day 7, which implies that chronic effects of parenteral arginine and/or the L-NAME infusion were observed. Regarding the effects of nutrition support, we believe that the effects of nutrition support on the immune responses would be more observable in a chronic phase than in an acute phase. Third, subacute peritonitic rats with oral feeding were not included in this study. As the aim of this study was to investigate the effects of parenteral arginine and/or L-NAME on immunity in subacute peritonitis, we believe that the current study design fulfilled the purpose of the present study. Fourth, to truly understand the immune functions of T cells and monocytes/macrophages, intracellular protein and CD markers can be used to determine which cells are proliferating and producing intracellular cytokines.

### Conclusion

The present study confirmed that parenteral feeding and subacute peritonitis may affect systemic and local immune systems, as shown in our elevated circulating WBC, spleen weights and splenocyte numbers. In addition, our findings indicated that parenteral arginine at approximately 3.24% of total calories may alleviate the subacute peritonitis-induced decreases in body weight gain without causing extensive NO production, and it may have potentials in activating humeral immunity and inactivating cellular immunity. For instance, parenteral arginine significantly increased B-leukocyte population, decreased numbers in cytotoxic T-splenocytes, NKT-leukocytes, and NKT-splenocytes, and alleviated IFN-γ production of T-splenocytes. In contrast, chronic L-NAME infusion may elevate inflammatory responses of leukocytes and eliminate splenocytic immunities, as evidenced by the increases in IL-6 and nitrate/nitrite productions of leukocytes and the decreases in splenocytic proliferation, the NKT-leukocyte population, IFN-γ productions of in leukocytes and splenocytes, and TNF-α production in splenocytes. The decreased serum nitrate/nitrite and increased B-leukocyte population levels in the subacute peritonitic rats with the combination treatment were mainly contributed by parenteral arginine.

In conclusion, parenteral arginine may attenuate parenteral nutrition- and peritonitis-associated immune impairment; however, L-NAME infusion may augment pro-inflammatory responses by leukocytes and eliminate pro-inflammatory and T-helper 1 responses by splenocytes in parenteral-fed rats with subacute peritonitis. These findings imply that parenteral arginine supplementation at approximately 3.24% of the total calories may have a therapeutic use in improving immune responses, as well as in attenuating inflammatory responses in peritonitic patients.
